# Placental transfer of third-generation antiepileptic drugs: in vivo lacosamide case study and in vitro investigation of transporter inhibition by lacosamide and perampanel

**DOI:** 10.1186/s40780-025-00537-z

**Published:** 2026-01-24

**Authors:** Ayami Ueda, Ayako Furugen, Ayako Nishimura, Takeshi Umazume, Ryoichi Aoyagi, Keisuke Okamoto, Katsuya Narumi, Hinata Ueda, Masaki Kobayashi

**Affiliations:** 1 Laboratory of Clinical Pharmaceutics & Therapeutics, Division of Pharmasciences, Faculty of Pharmaceutical Sciences, Hokkaido University, Kita-12-jo, Nishi-6-chome, Kita-ku, Sapporo, 060-0812 Japan; 2 Division of Healthcare Innovation in Pharmacy, Keio University Faculty of Pharmacy, 1-5-30 Shibakoen, Minato-ku, Tokyo, 105-8512 Japan; 3 Department of Pharmacy, Hokkaido University Hospital, Kita-14-jo, Nishi- 5-chome, Kita-ku, Sapporo, 060-8648 Japan; 4 Department of Obstetrics, Hokkaido University Hospital, Kita-14-jo, Nishi- 5-chome, Kita-ku, Sapporo, 060-8648 Japan

**Keywords:** Antiepileptic drugs, Lacosamide, Perampanel, Pregnancy, Placenta, Syncytiotrophoblast, UPLC/MS/MS, Efflux transporter

## Abstract

**Background:**

Antiepileptic drugs must be continually used to control seizures in pregnant people with epilepsy. Lacosamide (LCM) and perampanel (PER) are third-generation antiepileptic drugs that were introduced to clinical practice in 2008 and 2012, respectively. LCM and PER are increasingly being prescribed. However, little information is available regarding the safety or placental transfer of LCM and PER during pregnancy. We investigated the placental permeability of LCM in vivo and the effects of LCM and PER on efflux transporters in human placental cells in vitro.

**Methods:**

Clinical umbilical cord and maternal plasma samples were collected from a woman who had been receiving a regular oral regimen of 100 mg LCM twice daily. The LCM concentrations in the samples were quantified using ultra-high-performance liquid chromatography/tandem mass spectrometry. Accumulation assays were conducted using in vitro syncytiotrophoblast models to determine the effects of LCM and PER on the functions of efflux transporters, including breast cancer resistance protein (BCRP/*ABCG2*), multidrug resistance proteins (MRPs/*ABCCs*), and P-glycoprotein (P-gp/*ABCB1*).

**Results:**

The LCM concentrations in the maternal plasma and umbilical cord plasma samples were 4.02 µg/mL (predelivery trough level) and 3.22 µg/mL (3.5 h after last dose), respectively. The LCM concentration in the umbilical cord plasma was near the lower end of the maternal therapeutic range, and the fetal-to-maternal plasma concentration ratio likely remained below 1.0. Therapeutic concentrations of LCM and PER did not increase the accumulation of fluorescent substrates of the efflux transporters in the in vitro assays. In contrast, high LCM doses increased the accumulation of BODIPY™ FL prazosin, a BCRP substrate, whereas PER did not affect the accumulation of any substrate.

**Conclusions:**

LCM was transferred to the fetus at concentrations close to the lower end of the therapeutic range, suggesting that fetal plasma levels did not exceed the maternal levels. High LCM concentrations inhibited BCRP function in vitro, whereas PER did not inhibit major placental efflux transporters.

## Background

Epilepsy is among the most common neurological disorders worldwide. Approximately 50% of women with epilepsy are of childbearing age [1]. Antiepileptic drug (AED) use continues during pregnancy to control seizures, although fetal exposure to some AEDs, such as valproate, poses some risks to fetal development, potentially leading to malformations and reduced cognitive abilities [2, 3].

Levetiracetam and lamotrigine are the two most commonly prescribed AEDs for pregnant people with epilepsy [4]. Lacosamide (LCM) is a third-generation AED that was first approved by the European Medicines Agency (EMA) and the U.S. Food and Drug Administration (FDA) in 2008 and has been used worldwide to treat focal-onset seizures [5]. LCM is currently used as monotherapy for patients with focal-onset seizures in clinical practice [6]. LCM selectively enhances the slow inactivation of voltage-gated sodium channels [5]. Perampanel (PER) is another third-generation AED that was first approved by the EMA and FDA in 2012 for the adjunctive treatment of partial-onset seizures [7]. PER is a noncompetitive alpha-amino-3-hydroxy-5-methyl-4-isoxazolepropionic acid receptor antagonist [7]. Although newer AEDs are generally characterized by fewer clinically relevant drug–drug interactions and improved tolerability profiles, information on the safety of LCM or PER during pregnancy is scarce. The proportions of LCM and PER prescriptions for pregnant people in Japan has increased [8] but were low in 2020, at 3.1% and 2.8%, respectively. Data are insufficient on the safety of LCM and PER use in pregnant women, which may be why their use remains low in this population. Furthermore, the placental permeabilities of LCM and PER have not been fully evaluated.

Efflux transporters, such as breast cancer resistance protein (BCRP/*ABCG2*), multidrug resistance-associated protein 2 (MRP2/*ABCC2*), and P-glycoprotein (P-gp/*ABCB1*), are located in the human placenta and protect fetuses from exposure to foreign matter [9]. Importantly, placental ATP-binding cassette (ABC) transporters substantially modulate fetal drug exposure, and the inhibition or functional alteration of these transporters can increase transplacental transfer. Human epidemiological studies have reported that the concomitant use of substrates or inhibitors of placental P-gp is associated with an increased risk of congenital anomalies, supporting the concept that the inhibition of placental efflux transporters may compromise the fetal protective barrier [10, 11]. In addition to xenobiotics, placental ABC transporters regulate the distribution of a wide range of physiologically important substrates including steroid hormones, glucocorticoids, bile acids, and prostaglandins, thereby contributing to normal placental and fetal development [12]. Consequently, dysregulation of efflux transporter activity may affect not only fetal drug exposure but also the placental handling of endogenous substrates. Therefore, whether LCM and PER are substrates or inhibitors of efflux transporters in the human placenta must be determined to understand the effects of these drugs on the fetus. Zhang et al. performed concentration equilibrium transport assays using P-gp overexpressing cells, showing that LCM is a potential substrate for P-gp. However, an assay using human colon carcinoma Caco-2 cells indicated that passive diffusion plays a major role in the overall transfer of LCM [13]. These findings suggest that results obtained from transporter overexpression systems should be interpreted with caution when extrapolating to specific organs. Gonçalves et al. [14] reported that 2.5 to 75 µM LCM inhibits BCRP in MDCK-II cells transfected with human ABCG2 (MDCK-BCRP), independent of concentration. According to the Japanese Regulatory Interview Form (8th edition), PER is not a substrate of P-gp or BCRP but exhibits weak inhibitory activity against these transporters. Moreover, PER is not a substrate for organic anion transporters (OAT1, OAT2, OAT3, and OAT4) or organic cation transporters (OCT1, OCT2, and OCT3). However, it exerts inhibitory effects on OAT1, OAT3, OCT1, and OCT3. In contrast, PER increases the transport activity mediated by OAT2. PER is neither a substrate nor an inhibitor of OATP1B1 or OATP1B3. However, these data were derived from overexpression systems or non-placental models, and their functional relevance to the human placenta remains unclear. Furthermore, other efflux transporters have not been evaluated. Therefore, direct evaluation using a placenta-relevant model is necessary to assess the potential effects of LCM and PER on placental efflux transporter function.

Human trophoblast stem (TS) cells from cytotrophoblast (CT) cells of the early placenta or blastocytes (CT-derived TS: TS^CT^ or blastocyst-derived TS: TS^blast^) were used as tools to identify human trophoblast development and function [15]. TS cells differentiate into syncytiotrophoblasts (STs), and a layer of ST cells plays a crucial role in the human blood–placental barrier. We previously analyzed data on transporter expression in TS^CT^-derived ST (ST-TS^CT^) cells. The results described the expression of various efflux transporters, such as BCRP, MRP2, and P-gp, as well as their functions in cells [16]. In this study, ST-TS^CT^ cells were used in an in vitro model to determine whether LCM and PER affect efflux transporter expression in the human placenta. This has not been previously investigated using trophoblast cells.

In the present study, we investigated the placental permeability of LCM in vivo to obtain information regarding LCM use during pregnancy. Furthermore, we investigated the effects of LCM and PER on the function of efflux transporters in human placental cells in vitro.

## Materials and methods

### Chemicals

LCM, deuterated LCM (LCM-d_3_), and PER were purchased from Toronto Research Chemicals (Toronto, ON, Canada). HPLC-grade methanol was obtained from FIJIFILM Wako Pure Chemical Corporation (Osaka, Japan). HPLC-grade aqueous ammonium acetate solution was purchased from Nacalai Tesque (Kyoto, Japan). Pooled plasma samples from healthy donors were obtained from Cosmo Bio (Tokyo, Japan). MK571 and Rhodamine123 were purchased from Cayman Chemical (Ann Arbor, MI, USA), and Ko143 was purchased from Abcam (Cambridge, United Kingdom). We obtained 5(6)-carboxy-2’,7’-dichlorofluorescein diacetate (CDFDA) from Enzo Life Sciences Inc. (Farmingdale, NY, USA); elacridar was purchased from Wako (Tokyo, Japan). BODIPY™ FL prazosin was purchased from Thermo Fisher Scientific (Waltham, MA, USA).

### Quantifying LCM concentration in human plasma using UPLC/MS/MS

This study was approved by the Ethics Committee of the Hokkaido University Hospital (020–0139). The volunteer received a detailed explanation of this study and freely provided informed consent to participate. The volunteer was a woman admitted to the Obstetrics Department of Hokkaido University Hospital. The woman was orally administered LCM twice daily (100 mg × 2). The maternal plasma and umbilical cord plasma samples were collected 2.9 h before (trough level) and 3.5 h after oral LCM administration. Umbilical cord plasma samples were collected after delivery. The samples were stored at − 30 °C until analysis. The LCM concentrations in the clinical samples were quantified using UPLC/MS/MS, as previously described [17]. The calibration range was 0.5–100 ng/mL. As the LCM concentrations exceeded the range, the samples were diluted 100-fold with drug-free plasma.

### Cell culture

Human trophoblast stem (TS^CT^; CT29, RCB4937) cells derived from human CTs were purchased from Riken Cell Bank (Saitama, Japan). The TS^CT^ cells were cultured following a previously reported protocol [15]. The TS^CT^ cells were cultured in a collagen-IV-coated flask with TS medium, which consisted of Dulbecco’s modified Eagle’s medium (DMEM)/F12 (Wako) supplemented with 0.1 mM 2-mercaptoethanol, 0.2% fetal bovine serum, 0.5% penicillin–streptomycin, 0.3% fatty acid free bovine serum albumin, 1% ITS-X supplement, 1.5 µg/mL L-ascorbic acid, 50 ng/mL epidermal growth factor, 2 µM CHIR99021, 0.5 µM A83-01, 1 µM SB431542, 0.8 mM valproic acid, and 5 µM Y27632. The accumulation assay was performed by coating 48-well plastic plates with collagen IV, which were seeded with TS^CT^ cells at 5.0 × 10^4^ cells/well. The cells were cultured for 72 h at 37 °C in 5% CO_2_. TS^CT^ cell differentiation into ST-TS^CT^ cells was induced by replacing the culture medium with ST medium, which consisted of DMEM/F12 supplemented with 0.1 mM 2-mercaptoethanol, 0.5% penicillin–streptomycin, 0.3% bovine serum albumin, 1% ITS-X, 2.5 µM Y27632, 50 ng/mL epidermal growth factor, 2 µM forskolin, and 4% KnockOut™ Serum Replacement. The cells were cultured for 72 h as previously described [17]. TS^CT^ cell differentiation into ST-TS^CT^ cells under these conditions was confirmed in our previous study [18]. ST-TS^CT^ cells were used for the accumulation assays.

### Accumulation assay

An accumulation assay was conducted as previously described [16]. Fluorescent substrates (0.5 µM BODIPY™ FL Prazosin for BCRP, 0.5 µM CDFDA for MRPs, and 2 µM Rhodamine123 for P-gp) were used. CDFDA is uptaken by cells and intracellularly hydrolyzed to fluorescent CDF, a substrate for MRPs.

The inhibitory effects of LCM on the function of efflux transporters were examined by incubating the cells with different LCM concentrations. The LCM concentrations were calculated based on the clinical plasma concentration. The reference LCM concentration range in plasma is 4–40 µM [19] or 8.8–80 µM [20]. The assays were conducted at 10 µM (2.5 µg/mL), 20 µM (5.0 µg/mL), or 40 µM (10 µg/mL) LCM according to these data, which are within the therapeutic range. In addition, we investigated the effects of high LCM doses of 100 µM (25 µg/mL), 200 µM (50 µg/mL), and 400 µM (100 µg/mL). The reference PER concentration range in plasma is 0.14–1.1 µM [21] or 0.52–2.8 µM [22]. We tested the effects of therapeutic PER concentrations of 0.6 µM (209 ng/mL), 1.2 µM (419 ng/mL), and 1.8 µM (628 ng/mL) and at a high dose of 50 µM (17.5 µg/mL). We used 10 µM Ko143 (a BCRP inhibitor), 15 µM of MK571 (an MRP inhibitor), and 5 µM of elacridar (a P-gp inhibitor) as positive controls.

The cells were preincubated with or without the tested drugs for 30 min, and the buffer was aspirated once again. The cells were incubated with transport buffer containing fluorescent substrates with or without the tested drugs. The cells were then incubated for 60 min at 37 °C. The buffer was removed, and the cells were rinsed with ice-cold buffer three times and then were lysed with 200 µL of 0.1% Triton X-100/0.2 N NaOH. The accumulation of fluorescent substrates was measured on a plate reader at 485/528 nm for BODIPY™ FL Prazosin, 485/538 nm for CDF, and 485/535 nm for Rhodamine123. The fluorescence intensity was normalized to the protein level, which was determined using a Pierce^®^ BCA Protein Assay Kit (Thermo Scientific, Rockford, IL) according to the provided instructions.

### Statistical analysis

The experiments were repeated at least three times, as described in Section “Accumulation assay”. The data are presented as the mean ± standard error (SE) of independent experiments. Student’s t-test was used to determine the significance of the differences between groups. The significance of differences among three or more groups was determined using one-way analysis of variance (ANOVA), followed by Dunnett’s test. Statistical significance was set at *p* < 0.05.

## Results

### LCM concentrations in clinical samples

Clinical umbilical cord and maternal plasma samples were collected from a woman who had been orally administered LCM twice daily (100 mg ×2). The patient was not concomitantly taking any other drug. The patient first experienced seizures at twenties, several months prior pregnancy. The patient was sent to a hospital with status epilepticus. LCM and PER were prescribed to control the seizures, and no further seizures occurred. This child was the first delivery for the patient. PER was discontinued after pregnancy onset, and only LCM was administered during pregnancy. The female child was delivered vaginally at 36 weeks without any complications at the time of birth and weighed less than 2500 g. The Apgar scores 1 and 5 min after birth were within the normal range. The neonate exhibited mild somnolence as a symptom of drug withdrawal. In addition, neonatal jaundice was observed a few days after delivery and resolved within several days. No developmental delay was noted at the one-month checkup.

**Fig. 1:**
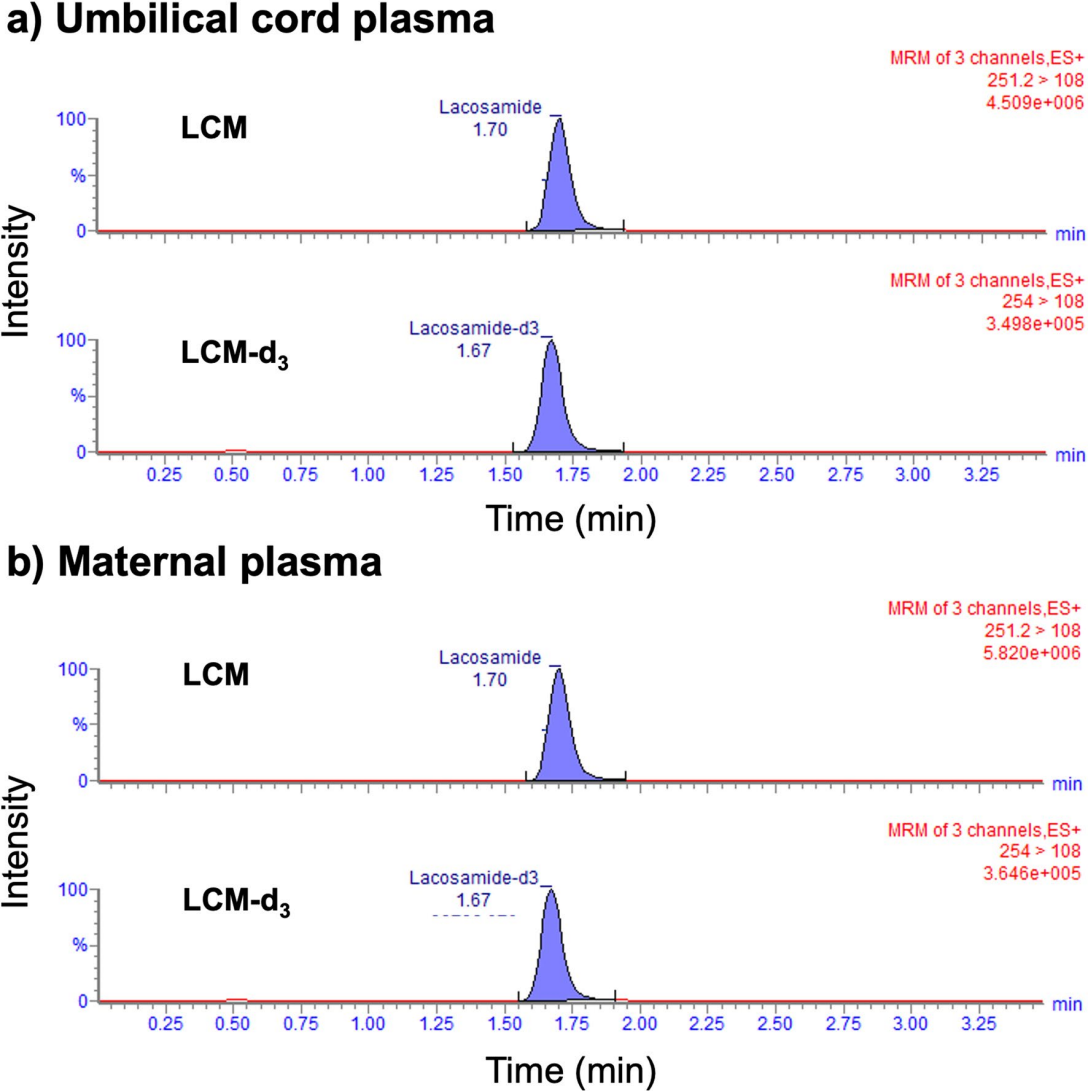
Chromatograms of lacosamide (LCM) and internal standard (IS: LCM-d_3_) in umbilical cord plasma (**a**) and maternal plasma (**b**)

The maternal and umbilical cord plasma samples were collected 2.9 h before (predelivery trough level) and 3.5 h (at time of birth) after oral LCM administration. Figure 1 shows that LCM was detected in the umbilical cord and maternal plasma, respectively, without any interfering peaks. The LCM concentrations in the umbilical cord and maternal plasma samples were calculated as 3.22 and 4.02 µg/mL, respectively (Table 1).

**Table 1:** Concentrations of lacosamide (LCM) in maternal and umbilical cord plasma samples

	Oral LCM administration	Time since last dose (h)	Supplemental information	Maternal concentration (µg/mL)	Umbilical cord concentration (µg/mL)
Day of delivery		**− 2.9**	Trough level	4.02	
	100 mg	0		-	
		**3.5**	Delivery	-	3.22
	100 mg	12		-	
Day 3 postpartum	100 mg	**0**		3.78	
		**1**		4.68	
		**2**		6.14	
	100 mg	12		-	

“-” indicates not measured

### Effects of LCM and PER on efflux transporter function in vitro

**Fig. 2:**
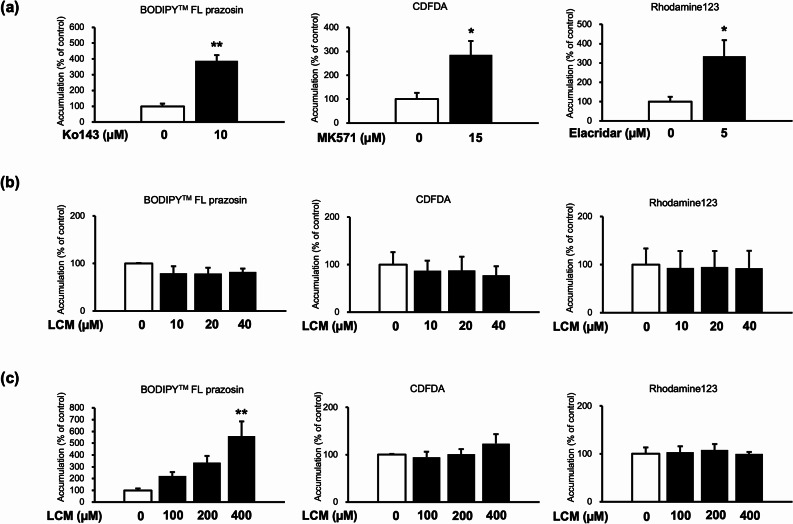
Effects of lacosamide (LCM) on the accumulation of BODIPY™ FL prazosin, 5(6)-carboxy-2′,7′-dichlorofluorescein diacetate (CDFDA), and Rhodamine123 in ST-TS^CT^. (**a**) Effects of the inhibitors (Ko143: 10 µM, MK571: 15 µM, and elacridar: 5 µM) as positive controls. (**b**) Effects of therapeutic LCM concentrations (10, 20, and 40 µM). (**c**) Effects of high LCM concentrations (100, 200, and 400 µM). The accumulation of fluorescent substrates was measured using a plate reader. Substrate accumulation was normalized to cell protein levels. Data are reported as the percentage of control (0 µM). Three independent experiments were performed in triplicate, and values are reported as mean ± SE. Significant differences in accumulation from that of control are indicated by asterisks (* *p* < 0.05, ** *p* < 0.01)

Accumulation assays using typical fluorescent substrates were conducted to investigate whether LCM affected the activity of efflux transporters BCRP, MRP, and P-gp in ST-TS^CT^ cells. BCRP function was assessed using BODIPY™ FL prazosin. For Ko143 (a BCRP inhibitor, positive control), the accumulation of BODIPY™ FL prazosin significantly increased compared to that of the control (*p* < 0.01, Fig. 2 (a)), indicating its inhibitory effect on BCRP transporter. In contrast, BODIPY™ FL prazosin accumulation did not significantly differ among the therapeutic LCM concentration (Fig. 2 (b)). The MRP function was assessed using CDFDA. For MK571 (an MRP inhibitor, positive control), the accumulation significantly increased compared to that of the control (*p* < 0.05, Fig. 2 (a)), while that of the LCM at all concentrations did not show statistically significant differences (Fig. 2 (b)). P-gp function was assessed using Rhodamine123. For elacridar (a P-gp inhibitor, positive control), the accumulation significantly increased compared to that of the control (*p* < 0.05, Fig. 2 (a)). The differences between the LCM and control groups were not significant at any therapeutic LCM concentration (Fig. 2 (b)). Thus, the therapeutic LCM concentration did not inhibit the efflux transporters.

The effects of high-dose LCM on efflux transporters are shown in Fig. 2(c). The accumulation of BODIPY™ FL prazosin increased with LCM concentration, and the increase in accumulation was significant at 400 µM (*p* < 0.01, Fig. 2(c)).

**Fig. 3:**
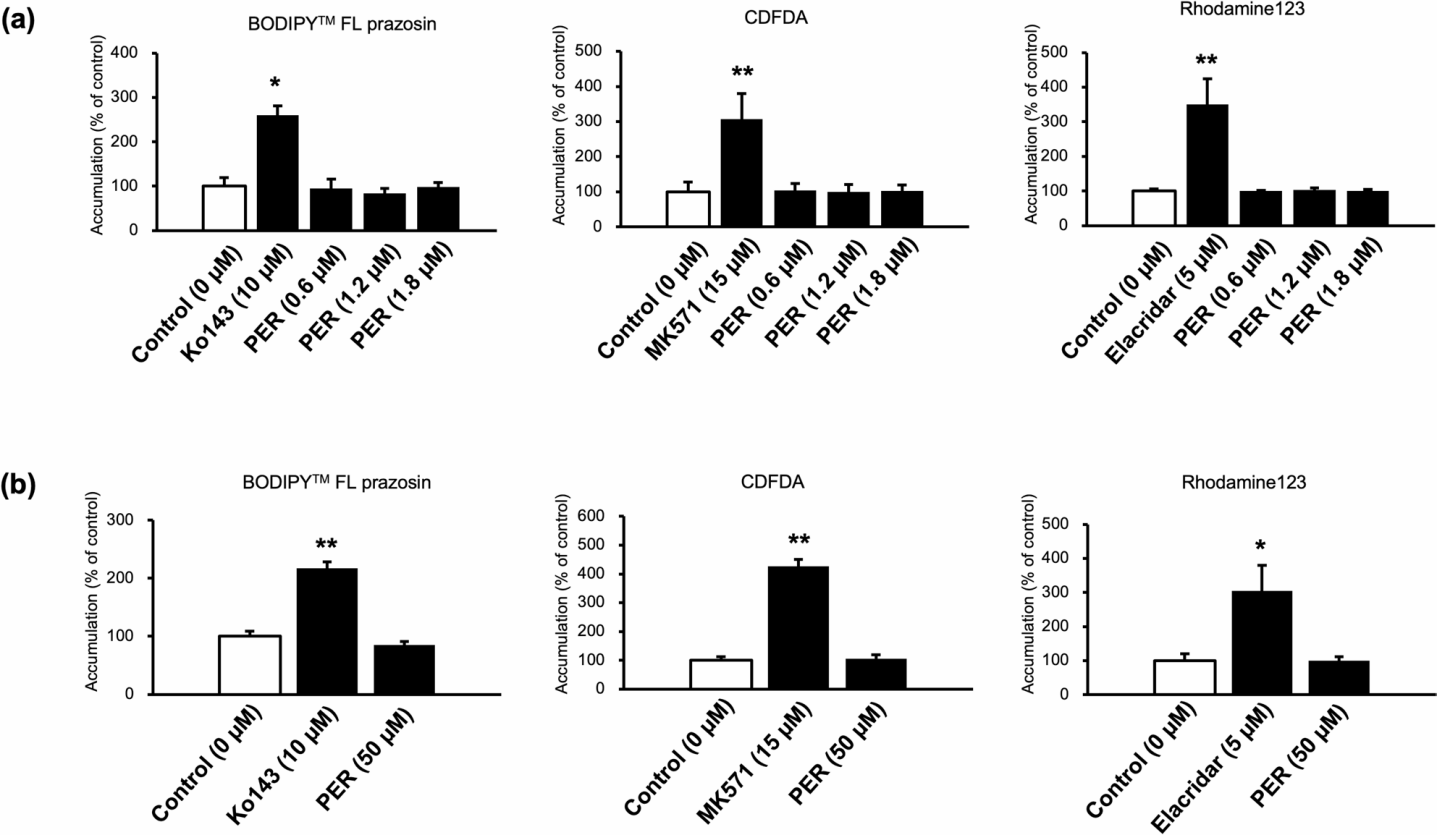
Effects of perampanel (PER) on accumulation of BODIPY™ FL prazosin, 5(6)-carboxy-2′,7′-dichlorofluorescein diacetate (CDFDA), and Rhodamine123 in ST-TS^CT^. (**a**) Effects of therapeutic PER concentrations (0.6, 1.2, and 1.8 µM). (**b**) Effects high PER concentration (50 µM). The accumulation of fluorescent substrates was measured using a plate reader. Substrate accumulation was normalized to cell protein levels. Data are reported as the percentage of control (0 µM). Three independent experiments were performed in triplicate, and values are shown as mean ± S. E. Significant differences in accumulation compared with that of the control are indicated by asterisks (* *p* < 0.05, ** *p* < 0.01)

The effects of PER on the function of efflux transporters are shown in Fig. 3. PER did not significantly increase the accumulation of BODIPY™ FL prazosin, CDFDA, or Rhodamine123 at any therapeutic concentration Fig. 3(a). Furthermore, BODIPY™ FL prazosin, CDFDA, and rhodamine123 accumulation was not affected by high PER doses Fig. 3(b).

## Discussion

The number of prescriptions of third-generation AEDs is expected to increase in the future owing to their novelty and because AED use must continue for pregnant patients with epilepsy. However, little information is available regarding the safety of LCM and PER during pregnancy [8]. In this study, we investigated the placental permeability of LCM in vivo and the effects of LCM and PER on efflux transporters in human placental cells in vitro.

We measured the in vivo LCM concentrations in clinical umbilical cord and maternal plasma samples collected from a pregnant woman who had been orally administered LCM. The LCM concentration in the maternal plasma was 4.02 µg/mL, which is within the reference range for LCM (2.5 to 10 µg/mL) [19, 20]. The patient had been receiving a regular oral regimen of LCM prior to delivery, and the plasma concentrations had reached a steady state. Steady-state plasma concentrations were achieved on day 3 following the oral administration of 200 mg LCM twice daily for 7 days, with a reported time to maximum concentration (Tmax) of 2.0 h (range: 2.0–3.0) and a terminal half-life (T1/2) of 13.91 ± 1.25 h on day 7, according to a study on healthy male adults [23]. The clinical records of the volunteer in this study showed that the LCM concentration in the umbilical cord plasma (collected at the time of delivery) 3.5 h after the last LCM dose was 3.22 µg/mL. In contrast, maternal plasma was collected on the same day, 9.1 h after the last LCM dose; the concentration was 4.02 µg/mL, likely reflecting a trough level. Additional maternal plasma samples were obtained on postpartum day 3, with LCM concentrations of 3.78 µg/mL at trough, 4.68 µg/mL at 1 h, and 6.14 µg/mL at 2 h after the dose (Table 1). Although these findings are based on limited sampling time points and simplified assumptions, the data suggest that the umbilical cord concentration of LCM was lower than the maternal plasma concentration and approximately corresponded to the lower limit of the therapeutic range. Berman et al. [24] reported that the ex vivo fetal-to-maternal ratio of LCM concentration after 180 min of human placental perfusion was approximately 1.0. These findings suggest that LCM does not preferentially accumulate in the fetus.

Our in vitro study demonstrated that LCM did not inhibit efflux transporters at therapeutic concentrations. However, high-dose LCM could inhibit BCRP. Gonçalves et al. [14] reported that 2.5–75 µM (0.63–18.8 μg/mL) LCM inhibited BCRP in BCRP-overexpressing cells, leading to an increase in the intracellular accumulation of Hoechst33342, another potential BCRP substrate. Since the assay in the present study was conducted at the endogenous expression level of BCRP in ST-TS^CT^ cells, it is possible that only high concentrations of LCM exhibited a concentration-dependent inhibitory effect on BCRP. This result shows that the effects of BCRP might be considered when the LCM concentration in the maternal plasma is high. A variety of clinically used drugs from different therapeutic classes have been reported to be substrates of BCRP. Antiseizure medications are often co-administered in clinical practice, although monotherapy is generally preferred during pregnancy. If high maternal plasma concentrations of LCM clinically inhibit placental BCRP, fetal exposure to co-administered BCRP-substrate antiseizure medications (e.g., lamotorigine [25]) may increase beyond the expected levels. In addition, BCRP is known to transport endogenous substrates such as steroid hormones [12]; therefore, inhibition of placental BCRP could theoretically influence the fetal microenvironment. However, the clinical relevance of these effects remains unclear and warrants further investigation. In this study, LCM did not affect the P-gp function. This finding contradicts that of Zhang et al. [13], as the concentration equilibrium transport assays using P-gp-overexpressing cells indicated that LCM may be a suitable substrate for P-gp. Our assay was conducted at endogenous P-gp levels in ST-TS^CT^ cells, in which LCM may not have significantly affected the transport of P-gp substrates. The findings of Zhang et al. [13] supported this hypothesis by showing that although LCM could be a substrate of P-gp, the data obtained from the Caco-2 assay demonstrated that passive diffusion should play a major role in the overall effects of LCM [13]. Efflux transporters may contribute to limiting fetal exposure and need to be considered because the LCM concentrations in the umbilical cord plasma were comparable to or lower than those in the maternal plasma in this study. The accumulation assays demonstrated that PER did not affect efflux transporter accumulation at therapeutic or high concentrations. Little information is available on the mechanisms of placental transfer of PER. Our results show the efflux transporters do not contribute to the placental transfer of PER at therapeutic or high concentrations.

For LCM, reported reference plasma concentration ranges are 4–40 µM (1.0–10 µg/mL) and 8.8–80 µM (2.2–20 µg/mL) [19, 20]. Additionally, in our separate clinical case involving a postpartum woman, the maximum observed plasma LCM concentration was close to the upper limit of the reported reference range (data not shown). Based on these clinically observed concentrations and reported literature values, the concentrations of LCM used in the present in vitro study (25, 50, and 100 µg/mL) were selected to encompass the upper range of clinical exposure and extend beyond it. These high-exposure conditions were established from a toxicological evaluation perspective, considering potential situations, such as overdose, reduced drug clearance, inter-individual variability, pregnancy-related pharmacokinetic changes, and concomitant medications. For PER, reported reference plasma concentration ranges are 0.14–1.1 µM (0.049–0.38 µg/mL) and 0.52–2.8 µM (0.18–0.98 µg/mL) [21, 22]. As clinical data for PER at high exposure levels were not available in our study, a concentration of 50 µM (17.5 µg/mL) was selected to simulate a plausible maximum exposure scenario. However, as the clinical relevance of this concentration remains unclear, these results should be interpreted with caution. In this study, albumin or other binding proteins were not added to the incubation buffer because our primary objective was to evaluate the direct effects of drugs on placental transporters. For LCM, plasma protein binding is low (protein binding < 15%) [23]; thus, the difference between nominal and unbound concentrations is expected to be minimal and unlikely to affect the interpretation of transporter involvement. In contrast, PER is highly protein-bound (approximately 95–96%) [26]. Under protein-free conditions, the unbound fraction may be higher in vitro than that under physiological conditions, and nominal concentrations may, therefore, overestimate the clinically relevant unbound exposure. Future studies should incorporate protein binding–adjusted conditions, such as the addition of human serum albumin at physiological concentrations.

We previously reported that the expression levels of efflux transporters in ST-TS^CT^ cells are considered to be more closely related to placental cells than the traditional BeWo cells [16]. However, the expression of efflux transporters differs between ST-TS^CT^ cells and placental tissues. Furthermore, placental transporter expression varies across gestational stages [27] and such temporal changes cannot be fully evaluated in vitro. Further studies using placental tissues are necessary to validate the in vivo relevance of these findings.

Few case reports have been published on the use of LCMs during pregnancy. Zutshi et al. [28] reported that none of the infants whose mothers were administered LCM experienced teratogenic malformations at birth. An infant died at 5 months whose mother was administered 400 mg LCM per day; however, other factors, such as low birth weight, likely contributed to the death of the infant. Ylikotia et al. [29] reported that a mother who was administered 200 mg of LCM and 2000 mg of levetiracetam per day gave birth to an infant without any teratogenic malformations. Kitamura et al. [30] reported that a patient treated with oral LCM (400 mg/day) gave birth to a neonate without any congenital anomalies or neonatal drug withdrawal symptoms. Our results and the case report show that although LCM is transferred to the fetus via the placenta, the likelihood of LCM concentrating in the fetus is low. Additional studies are needed to determine the safety of LCM use during pregnancy. Alicino et al. reported that in a small case series of four pregnancies exposed to PER, no major congenital malformations or adverse birth outcomes were observed [31]; PER studies are also required.

Our study has a few limitations. First, we only measured the LCM concentration in one set of clinical samples of umbilical cord plasma and maternal plasma. This does not take a possible interindividual variety into account. Second, the maternal and umbilical cord plasma samples were not simultaneously collected; therefore, the exact fetal-to-maternal plasma concentration ratio could not be determined. Third, we conducted a series of accumulation assays that indicated the effect of a drug on efflux transporters and not the intracellular transport of the drug. In addition, we only evaluated the effects of the drugs on efflux and not influx transporter accumulation.

## Conclusions

We evaluated the placental transfer of a third-generation AED, LCM, in vivo in a case of a patient administered LCM during pregnancy. We investigated the effects of LCM and PER on the function of efflux transporters in vitro using the ST model. The in vivo studies suggested that, although LCM is transferred to the fetus via the placenta, the likelihood of the drug concentrating on the fetal side is low. To the best of our knowledge, this is the first report to describe the umbilical cord concentration of LCM in a clinical sample. In addition, we found that therapeutic LCM and PER concentrations had little effect on efflux transporter accumulation. However, high concentrations of LCM may affect BCRP function. Further studies are required to establish information regarding the use of LCM and PER during pregnancy.

## Data Availability

All data generated or analyzed during this study are included in this published article.

## Abbreviations

AED: Antiepileptic drug
ANOVA: One-way analysis of variance
BCRP: Breast cancer resistance protein
CDF: 5 (and 6)-carboxy-2,7-dichlorofluorescein
CDFDA: 5(6)-carboxy-2’,7’-dichlorofluorescein diacetate
CT: Cytotrophoblast
DMEM: Dulbecco’s modified Eagle medium
IS: Internal standard
LCM: Lacosamide
MRPs: Multidrug resistance proteins
PER: Perampanel
P-gp: P-glycoprotein
ST: Syncytiotrophoblast
TS: Human trophoblast stem
UPLC/MS/MS: Ultra-high-performance liquid chromatography/tandem mass spectrometry
